# Analysis of hardware complications and functional outcomes following sternoclavicular hook plate fixation for traumatic dislocations

**DOI:** 10.1016/j.xrrt.2026.100815

**Published:** 2026-07-09

**Authors:** Li Sheng, Youyou Zhu, Yaqing Wu, Feifei Wang, Bin Wang, Xiaoyong Sheng

**Affiliations:** a Hangzhou Geriatrics Hospital (Hangzhou First People's Hospital Chengbei District), Hangzhou, Zhejiang, China; b Taizhou Hospital of Zhejiang Province, Linhai, Zhejiang, China; c Department of Orthopaedics, Taizhou Hospital of Zhejiang Province, Enze Medical Center, Linhai, Zhejiang, China

**Keywords:** Sternoclavicular joint dislocation, Hardware failure, Joint stabilization, Post-operative outcomes, Complications, Shoulder function

## Abstract

**Background:**

This study evaluates the efficacy and complications associated with sternoclavicular (SC) hook plates in treating traumatic sternoclavicular joint (SCJ) dislocations.

**Methods:**

We retrospectively analyzed patients who underwent SC hook plate fixation for SCJ dislocations, focusing on hardware-related complications such as plate breakages and fixation failures. Clinical outcomes, functional recovery, and complications were assessed. Post-operative imaging, including X-rays and computed tomography scans, was used to evaluate joint reduction and hardware integrity. Functional outcomes were measured using the Constant Murley Shoulder Score and visual analog scale for pain.

**Results:**

Forty-six patients (mean age: 49.3 years; 44 males [95.7%]) were analyzed, with 14 patients (30.4%) categorized as having a high preinjury activity level (Tegner scale ≥6). The SC hook plates effectively stabilized the joint, as evidenced by a significant increase in the mean Constant Murley Shoulder Scores from 36.4 ± 4.8 pre-operatively to 86.6 ± 6.2 post-operatively and a decrease in visual analog scale scores from 7.2 ± 1.4 to 1.6 ± 0.4. Hardware-related complications occurred in 5 patients (10.9%): consisting of 4 cases (8.7%) of asymptomatic plate breakage and 1 case (2.2%) of fixation failure. In the complication group (n = 5), 80% (4/5) were activity-level patients, compared to 24.4% (10/41) in the non-complication group. High preinjury activity level identified as a potential risk factor for plate breakage (*P* = .018). However, this should be interpreted as a preliminary, hypothesis-generating finding rather than a definitive conclusion given the limited number of events.

**Conclusion:**

While SC hook plates are effective in restoring SCJ function, they are associated with a notable rate of fatigue-related hardware complications. These preliminary findings highlight the importance of prudent patient selection and suggest the hook plate functions best as a temporary stabilization solution.

Sternoclavicular joint (SCJ) dislocations are uncommon but clinically important injuries that present substantial challenges in trauma surgery because of the joint's proximity to vital mediastinal structures and its essential role in shoulder kinematics.^4^ While many anterior dislocations can be managed non-operatively, posterior dislocations and unstable anterior injuries often require surgical stabilization to restore function and prevent serious complications.^5^ Despite multiple described techniques, optimal operative management of unstable SCJ dislocations remains controversial.

The SCJ is the only true osseous articulation between the upper limb and the axial skeleton and functions as a highly mobile, triaxial joint.^2^ Physiological shoulder motion generates continuous multidirectional cyclic loading through the joint, creating a uniquely demanding biomechanical environment for internal fixation. Rigid implants applied across this articulation must therefore withstand repetitive stresses while maintaining stability, raising fundamental concerns regarding long-term mechanical durability.

Various surgical strategies have been reported,^10^^,^^18^ including ligament reconstruction, suture-based fixation, and adaptations of acromioclavicular implants, each with distinct advantages and limitations. Recently, a sternoclavicular (SC) hook plate specifically designed for SCJ fixation has become available, providing immediate rigid stabilization via a sternal hook–clavicular plate construct. However, despite its increasing clinical use, while recent studies have explored the application of hook plates in complex shoulder girdle injuries, including proximal clavicle fractures and mixed SC injuries, clinical evidence focused specifically on pure traumatic SCJ dislocations remains limited. Furthermore, the temporal and morphological patterns of implant fatigue—and their potential dissociation from final functional outcomes—have not been systematically characterized in this specific patient population.

Biological stabilization of the SCJ through capsuloligamentous healing typically evolves over several months following reduction.^1^ Once this process is complete, continued rigid fixation may no longer be required for joint stability and may instead expose the implant to cumulative fatigue loading,^19^ particularly in physically active patients. Under this paradigm, late implant breakage may represent a possible mechanical consequence of prolonged retention across a mobile joint rather than failure of initial surgical stabilization. We therefore hypothesized that the SC hook plate provides temporary mechanical stabilization during the biological healing phase and that patient activity level primarily accelerates fatigue accumulation rather than directly causing clinical failure.

Under this framework, we hypothesized that the SC hook plate would achieve reliable joint reduction and favorable functional recovery, but that its use would be associated with a specific complication profile, including hardware breakage, which might be influenced by identifiable clinical variables. Accordingly, the primary objective of the present study was to evaluate the clinical and radiographic outcomes of hook plate fixation for traumatic SCJ dislocations, characterize the incidence and nature of hardware-related complications, and investigate potential clinical factors, such as patient activity level, associated with these events. By clarifying these associations, this study aims to provide an initial clinical foundation to inform surgical strategy and evidence-based management of unstable SCJ dislocations.

## Methodology

### Study design

This retrospective cohort study was conducted at a single tertiary care institution. We consecutively analyzed 46 patients who received SC hook plate fixation for acute SCJ dislocations (within 3 weeks of trauma) between January 2015 and December 2022. This study was conducted in accordance with the principles of the Declaration of Helsinki and received approval from the institutional review board (approval number: kL20240919). Written informed consent was obtained from all participants.

Patients were enrolled in this study if they met one of the following inclusion criteria: (1) failure of closed reduction, (2) redislocation following initial repositioning, or (3) inability to tolerate prolonged immobilization (defined as localized pain with visual analog scale [VAS] >7 within 2 weeks non-responsive to non-steroidal anti-inflammatory drugs and weak opioids, or documented brace intolerance), thereby requiring surgical stabilization. These anterior cases underwent surgery for 3 reasons: gross instability characterized by immediate redislocation after successful closed reduction, irreducible dislocation caused by soft tissue or capsular interposition, and high functional demands that precluded conservative bracing. Stable anterior dislocations were initially managed conservatively, and surgery was only performed for patients with documented mechanical failure or high-risk features. All posterior SCJ dislocations were treated surgically due to the inherent risks of mediastinal injury and persistent joint instability.

Exclusion criteria were chronic SCJ injuries (duration >3 weeks), conservative management, or the presence of systemic comorbidities known to impair bone or soft-tissue healing, included uncontrolled diabetes mellitus, established systemic osteoporosis, chronic corticosteroid therapy, and renal insufficiency. Patient activity level was categorized based on the Tegner activity scale, where levels ≥6 were defined as high activity (eg, manual laborers or competitive athletes). This scale evaluates activity mainly by occupation and sports participation.

### Surgical technique

All procedures were performed by a single senior shoulder surgeon with >10 years of subspecialty experience. Following the induction of general anesthesia, the patient was placed in the supine position. An inverted L-shaped incision was made along the upper sternal midline. The periosteum over the affected side of the sternum, together with the sternocleidomastoid muscle insertion, was carefully elevated using a periosteal elevator.

### Joint reduction and fixation

**Figure 1:**
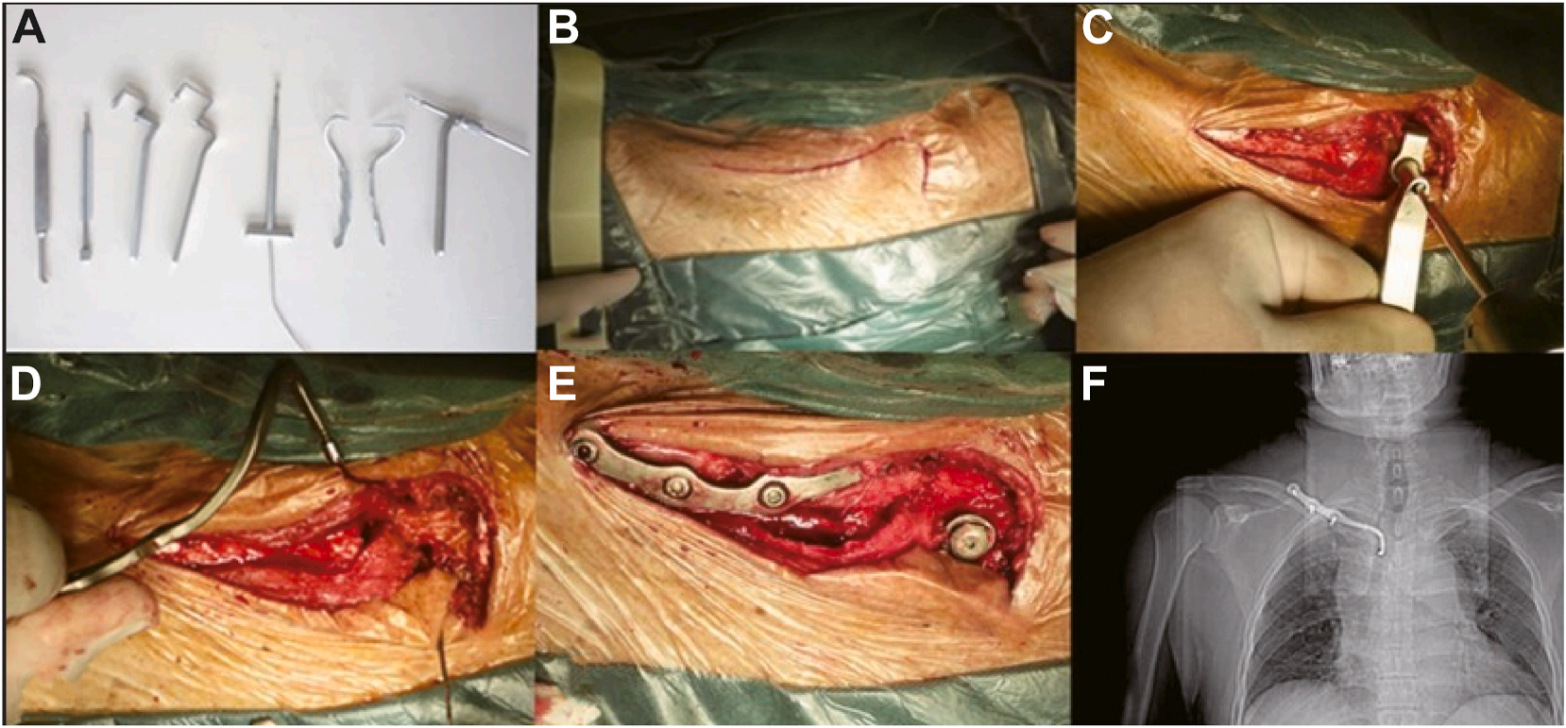
Schematic illustration of the treatment of anterior dislocation of the sternoclavicular joint (SCJ) in a 56-year-old man with a sternoclavicular (SC) hook plate. (**A**) SCJ plate and supporting tools. (**B**) Surgery incision shape. (**C**) Guide behind the sternum and drill holes. (**D**) SCJ plate passes through the sternum stem along the guidewire. (**E**) Plate fixes the SCJ. (**F**) X-ray shows that the plate fixes the SCJ.

**Figure 2:**
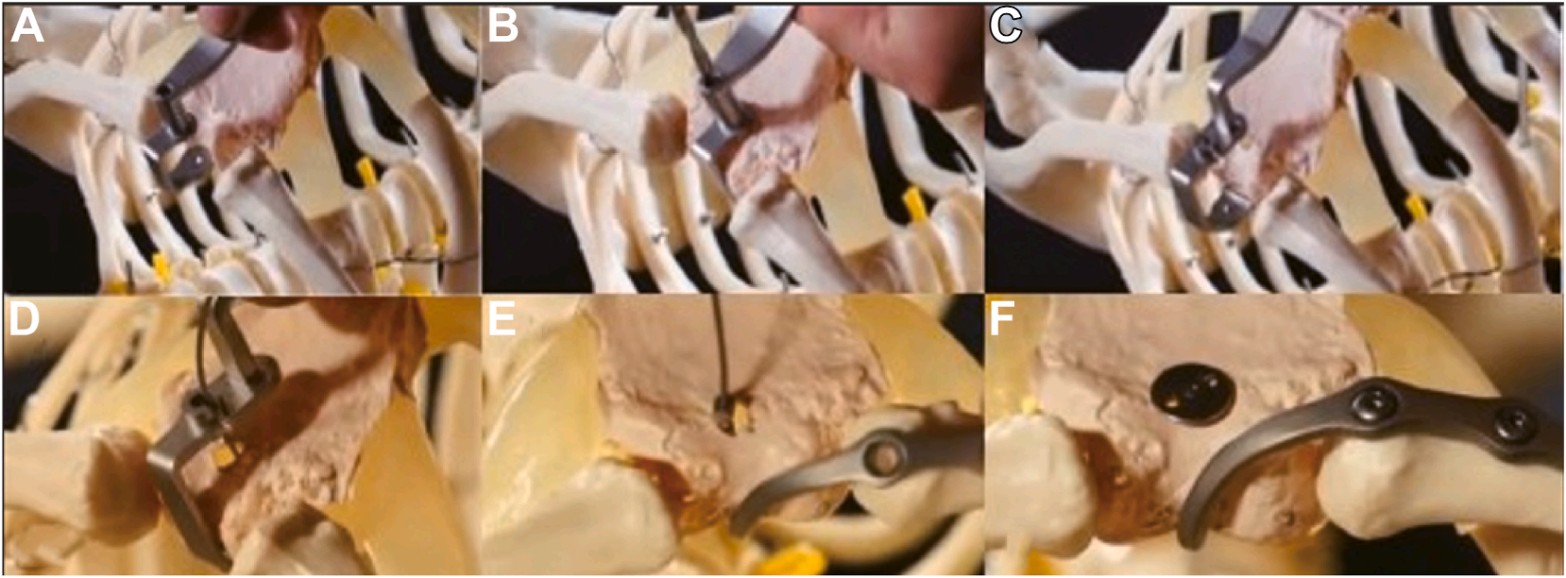
Simulation of sternoclavicular (SC) hook plate surgery on a mannequin. (**A**) Sternum drilling guide is placed immediately behind the sternal styloid. (**B**) Sternum styloid drilling is performed with a 4.2 mm diameter drill. (**C**) Sternum pulling guide is placed behind the sternum. (**D**) Cable head is connected to the guide. (**E**) Cable is connected to the hook end of the plate and is led out through the sternal foramen. (**F**) Sternoclavicular joint immobilization is completed.

All instruments, including a manufacturer-provided drill guide, were part of the standard SC hook plate system (Zhejiang CANWELL Medical Equipment Co.) (Figs. 1 and 2). After surgical exposure was achieved, a custom-designed drill guide was aligned along the sternal midline. Under fluoroscopic guidance, a 4.2-mm cannulated drill bit was used to create a bicortical tunnel, with depth meticulously controlled (20-25 mm) to prevent mediastinal penetration. A curved metallic retractor was positioned posterior to the sternum throughout the procedure to protect underlying vital structures.

Manual reduction of the SCJ was then achieved by applying inferior traction to the proximal clavicle while stabilizing the sternum. In cases of posterior dislocation, the retrosternal clavicle was first carefully reduced using elevators. The SC hook plate was subsequently applied. A cable passer was introduced through the sternal tunnel, engaged with the plate's hook cable, and tensioned to secure the hook within the tunnel. The plate was contoured to fit the superior clavicular surface, and 3 3.5-mm cortical screws (20-25 mm in length) were inserted after drilling and tapping. If residual instability (≥2 mm translation on stress views) was detected, a 1-2 mm shim washer was placed between the plate and clavicle to enhance compression. Final reduction and hardware position were confirmed intraoperatively with fluoroscopy.

### Closure and reinforcement

The joint capsule was repaired with non-absorbable sutures (Ethibond 2-0 Suture model), and the sternocleidomastoid muscle was reattached to its sternal origin with interrupted sutures. The subcutaneous layer was closed with absorbable sutures, and skin closure was achieved with staples or a running nylon suture.

### Intraoperative safety measures

Real-time fluoroscopy was used throughout to verify tunnel position, implant alignment, and joint reduction. The posterior sternal retractor was maintained during drilling and cable passage to prevent mediastinal injury. After fixation, stability was assessed through passive abduction to 90° and anteroposterior stress testing under fluoroscopy.

### Post-operative management

Patients were immobilized post-operatively using a triangular shoulder sling. A structured rehabilitation protocol was initiated at 2 weeks, involving graduated functional exercises with shoulder abduction and flexion limited to 90° for the first 6 weeks; strengthening exercises were commenced after 6 weeks. Vigorous physical activities were restricted for an additional 3 months to facilitate soft-tissue healing. Elective plate removal was scheduled between 6 and 12 months, contingent upon confirmed radiographic stability and clinical stability.

### Radiographic and clinical follow-up

A standardized surveillance protocol was implemented. All patients underwent immediate post-operative imaging (within 24 hours), including anteroposterior radiographs and computed tomography (CT), to verify reduction and hardware position. Serial follow-up included biplanar radiographs at 1, 3, 6, and 12 months post-operatively, with CT scans repeated at 6 and 12 months for cases with initial instability. All images were independently assessed by 2 musculoskeletal radiologists focusing on joint congruity and hardware integrity; discrepancies were resolved by consensus.

Clinical assessments were performed by an independent orthopedic clinical specialist who was blinded to the radiographic findings. Clinical assessments were conducted at 2 weeks, 1, 3, 6, and 12 months, using the Constant Murley Shoulder Score (CSS) and VAS. Beyond 24 months, patients underwent annual clinical or telemedicine reviews to monitor long-term outcomes and delayed complications.

### Data collection

Data were retrospectively extracted from electronic medical records and the hospital’s picture archiving and communication system (Carestream Health, Rochester, NY, USA) according to a prespecified protocol. A standardized data extraction form was used to collect patient demographics, injury characteristics, surgical details, and post-operative clinical courses. Data on demographics, injury details, surgical parameters, and post-operative outcomes (including CSS and VAS scores) were retrospectively extracted from electronic medical records and picture archiving and communication system and were recorded from scheduled clinical visits. Radiologic outcomes were assessed based on the formal reports of all follow-up imaging studies. To ensure data integrity, 2 researchers independently performed the initial data extraction. All information was then entered into a centralized database, and any discrepancies identified during a subsequent cross-checking process were resolved through consensus or by adjudication from a senior clinician.

### Statistical analysis

Statistical analyses were conducted using SPSS version 26.0 (IBM Corp., Armonk, NY, USA). Continuous variables (eg, CSS, VAS scores) are presented as mean ± standard deviation and were compared using the paired *t*-test or Wilcoxon signed-rank test depending on normality. To identify potential risk factors for hardware-related complications (including plate breakage and fixation failure), univariate analyses were performed. Specifically, categorical variables such as activity level (high vs. low/moderate), bone quality (normal vs. osteoporotic), and injury energy were analyzed using Fisher's exact test. Continuous risk factors, such as age and time to implant removal, were compared between the complication and non-complication groups using the Mann-Whitney U test due to the small size of the complication subgroup. A 2-tailed *P* value < .05 was considered statistically significant.

Categorical variables, including activity level and bone quality, were analyzed using Fisher's exact test. Due to the inherently limited sample size of the complication group (n = 5) relative to the non-complication group (n = 41), it must be noted that these statistical comparisons have restricted power. Consequently, all reported *P* values should be interpreted with caution and regarded as hypothesis-generating rather than definitive confirmatory findings.

## Results

### Patient demographics and clinical data

A total of 46 patients with traumatic SCJ dislocations were treated with SC hook plates between 2015 and 2022. The cohort had a mean age of 49.3 years (range: 28-73 years) and was predominantly male (44 males, 95.7%). The mean follow-up duration was 26.4 ± 8.2 months (range: 12-48 months). The majority of injuries resulted from high-energy trauma, with traffic accidents accounting for 44 cases and sports injuries for 2 cases (both occurred during competitive soccer matches, when the patient sustained a direct blow to the shoulder, resulting in an anterior dislocation). Anterior dislocations were observed in 42 patients (91.3%), while 4 cases (8.7%) were posterior dislocations. Regarding the severity of the initial injury, the mean pre-operative displacement of the proximal clavicle was measured as 24.8 ± 5.2 mm on axial CT scans, reflecting the high-energy nature of the traumatic dislocations in our series. Detailed demographic and baseline clinical characteristics are summarized in Table I.

**Table I:** Patient demographics, surgical data, and functional outcomes (n = 46)

Part I: Baseline demographics and surgical characteristics	
Variables	Value
Demographics	
Age (yrs), mean ± SD	49.3 ± 3.6
Gender (male/female), n (%)	44 (95.7%)/2 (4.3%)
High activity level (Tegner scale ≥6), n (%)	14 (30.4%)
Mean follow-up duration (mo)	26.4 ± 8.2 (range: 12-48)
Injury characteristics	
Dislocation direction (anterior/posterior), n (%)	42 (91.3%)/4 (8.7%)
Injury mechanism (traffic accident/sports), n (%)	44 (95.7%)/2 (4.3%)
Pre-operative displacement (mm), mean ± SD	24.8 ± 5.2
Surgical data	
Operative time (min), mean ± SD	65.2 ± 8.4
Estimated blood loss (mL), mean ± SD	46.2 ± 14.6

Part II: Pre-operative and post-operative functional outcomes			
Outcome measures	Pre-operative	Final follow-up	*P* value
CSS	36.4 ± 4.8	86.6 ± 6.2	<.05
VAS	7.2 ± 1.4	1.6 ± 0.4	<.05
ROM			
Forward flexion, °	65.2 ± 12.4	162.5 ± 8.2	<.05
Abduction, °	58.4 ± 10.6	158.2 ± 7.4	<.05
External rotation, °	22.5 ± 5.8	42.6 ± 6.2	<.05

*SD*, standard deviation; *CSS*, Constant Murley Shoulder Score; *VAS*, visual analog scale; *ROM*, range of motion.

Statistical significance: *P* < .05 was considered statistically significant. All comparisons between pre-operative and post-operative values were performed using the paired *t*-test or Wilcoxon signed-rank test.

While many anterior SCJ dislocations are candidates for non-operative management, the high proportion of anterior injuries (91.3%) in this surgical cohort reflects a specific subset of patients with high functional demands or severe instability. Beyond the general inclusion criteria, surgical intervention for these 42 anterior cases was specifically warranted based on the following clinical findings: (1) gross instability, defined as immediate and complete redislocation upon cessation of manual pressure or during gentle range-of-motion testing following a successful closed reduction; (2) irreducible dislocation, where soft-tissue interposition or capsular entrapment prevented anatomic alignment despite multiple closed attempts; and (3) high-demand profiles, specifically manual laborers and athletes whose professional obligations required a level of joint stability and early mobilization that conservative bracing could not reliably provide. In our practice, a “wait-and-see” approach was initially attempted in stable presentations, and only those with documented mechanical failure or high-risk profiles proceeded to hook plate fixation.

### Surgical and functional outcomes

All procedures were successfully completed. The mean operative time was 65.2 ± 8.4 minutes (range: 55-95), with an average estimated blood loss of 46.2 ± 14.6 mL. No intraoperative neurovascular injuries or early post-operative soft-tissue complications were observed.

Immediate post-operative imaging confirmed successful anatomic reduction and stable internal fixation in all patients. Functional outcomes demonstrated significant improvement at final follow-up. The CSS increased from a pre-operative value of 36.4 ± 4.8 to 86.6 ± 6.2 points (*P* < .05). The mean VAS score for pain decreased from 7.2 ± 1.4 to 1.6 ± 0.4 (*P* < .05). Furthermore, significant enhancements were noted across all planes of shoulder range of motion. Specifically, mean forward flexion improved from 65.2 ± 12.4° to 162.5 ± 8.2° (*P* < .05), mean abduction increased from 58.4 ± 10.6° to 158.2 ± 7.4° (*P* < .05), and mean external rotation increased from 22.5 ± 5.8° to 42.6 ± 6.2° (*P* < .05).

Radiographic evaluation performed within the first 24 hours post-operatively and at the 1-month follow-up evaluation showed no signs of hardware loosening or displacement. The actual mean timing for elective implant removal was 8.3 ± 2.0 months (range: 6-12 months) post-operatively. Specifically, the complication group underwent removal at a mean of 8.8 ± 1.7 months, while the non-complication group underwent removal at 8.2 ± 2.1 months (*P* = .560).

Although dynamic magnetic resonance imaging was not performed to assess soft-tissue healing, direct intraoperative visualization during the secondary surgery for hardware removal (including cases of plate breakage) confirmed robust fibrous healing and structural stability of the joint capsule in all instances. This intraoperative finding suggests that biological stability had been successfully restored prior to the occurrence of asymptomatic hardware failure.

### Complications and hardware issues

**Figure 3:**
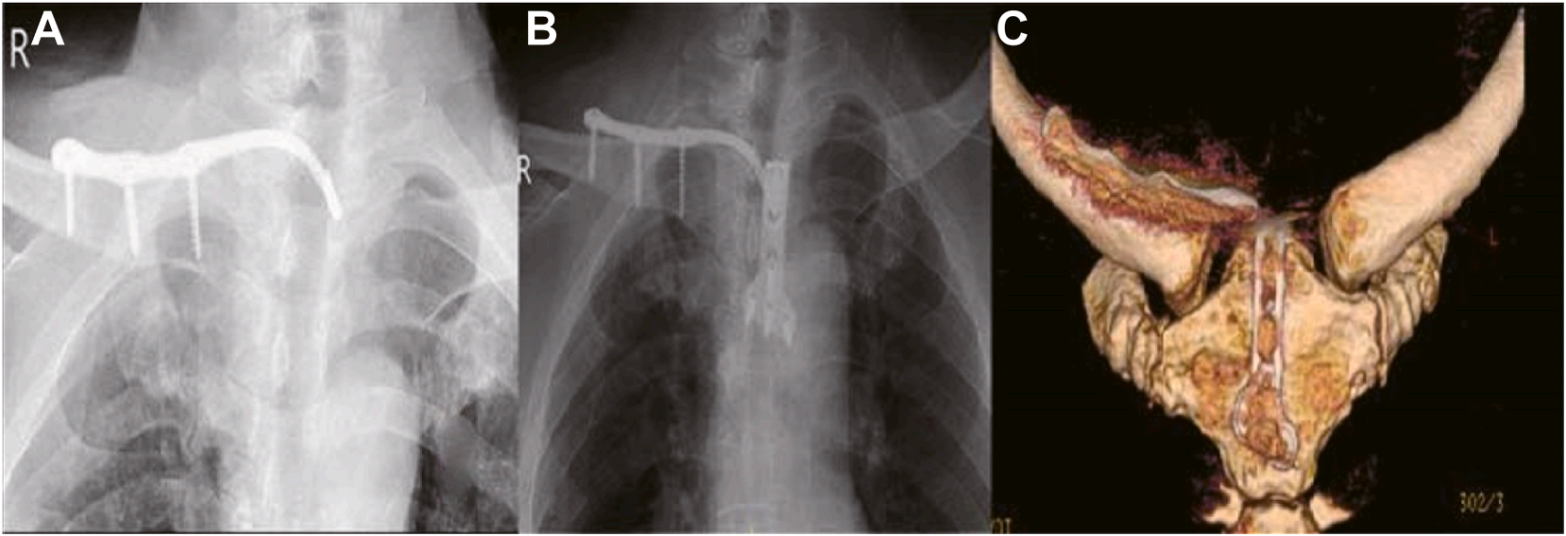
66-year-old male patient had internal fixation failure and redislocation. (**A**) X-ray of the SCJ fixation was loose on day 10 after surgery. (**B**) X-ray of the reoperation image. (**C**) Computed tomography (CT) 3-dimensional (3D) reconstruction of reoperation. *SCJ*, sternoclavicular joint.

**Figure 4:**
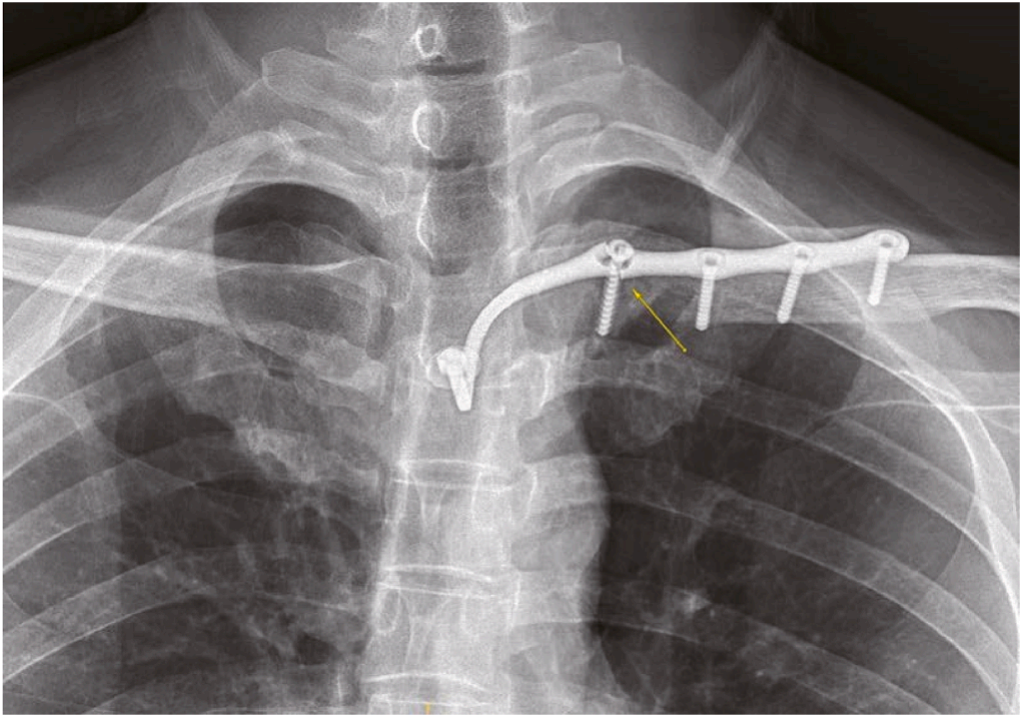
A 36-year-old male patient with an SC hook plate used for the treatment of anterior dislocation of the SCJ experienced plate breakage 7 months postsurgery, with no subsequent reoccurrence of SCJ dislocation. *SC*, sternoclavicular; *SCJ*, sternoclavicular joint.

Five patients (10.9%) experienced hardware-related complications during the follow-up period. One patient (2.2%), a 66-year-old male, developed fixation failure accompanied by redislocation within 10 days after surgery, necessitating revision operation (Fig. 3). In 4 additional cases (8.7%), asymptomatic plate breakage was identified during follow-up; all 4 cases of plate breakage occurred in patients who retained the implant for more than 6 months. A descriptive observation was that these patients tended to be younger (all aged less than 45 years), which aligned with the subsequent identification of high activity level as a significant risk factor. However, none of these instances resulted in loss of reduction or required immediate surgical intervention (Fig. 4) (Table II).

**Table II:** Clinical characteristics and outcomes of patients with hardware-related complications (n = 5)

Case no.	Age (yrs)/sex	Activity level (Tegner)	Bone quality	Dislocation direction	Complication type	Time to failure (mo)	Implant removal	Final CSS score
1	66/M	Low	Osteoporotic	Anterior	Fixation failure	< 0.5 (10 d)	Yes (revision)	72
2	28/M	High	Normal	Anterior	Plate breakage	7	Yes	88
3	35/M	High	Normal	Posterior	Plate breakage	9	Yes	92
4	38/M	High	Normal	Anterior	Plate breakage	8	Yes	86
5	43/M	High	Normal	Anterior	Plate breakage	11	Yes	90

*CSS*, Constant Murley Shoulder Score; *M*, male.

Activity level: Categorized based on the Tegner activity scale; “High” refers to a score ≥6. Fixation failure: In case 1, this occurred due to early redislocation within 10 days post-operatively. Plate breakage: In cases 2-5, breakage was asymptomatic and identified during routine follow-up; no loss of reduction was observed. Timing: All cases of plate breakage (cases 2-5) occurred in patients who retained the implant for more than 6 months.

### Descriptive observation of clinical outcomes

To evaluate the clinical impact of hardware integrity, we descriptively compared patients with asymptomatic plate breakage (n = 4) to those with intact hardware at the time of elective removal (n = 41). At the final follow-up, clinical scores appeared consistent between the 2 groups. The mean CSS in the plate breakage group was 89.0 ± 2.6, which was numerically similar to the 86.4 ± 6.4 observed in the intact group (*P* = .624). Similarly, the mean VAS score for the breakage group (1.5 ± 0.5) showed no clinical deviation from the intact hardware group (1.6 ± 0.4, *P* = .852) (Table III). These observations suggest that late mechanical breakage, occurring after the initial healing phase, did not result in a measurable loss of reduction or a decline in functional recovery in this small series.

**Table III:** Comparison of functional outcomes between patients with asymptomatic plate breakage and intact hardware

Outcome measure	Plate breakage group (n = 4)	Intact hardware group (n = 41)	*P* value
Constant Murley Score (CSS)	89.0 ± 2.6	86.4 ± 6.4	.624∗
VAS pain score	1.5 ± 0.5	1.6 ± 0.4	.852∗
Joint stability (reduction)	4/4 (100%)	41/41 (100%)	>.999†
Return to activity (mo)	4.2 ± 0.8	4.5 ± 1.2	.510∗

*CSS*, Constant Murley Shoulder Score; *VAS*, visual analog scale.

∗ Calculated using the Mann-Whitney U test.

† Calculated using Fisher's exact test. *P* > .05 indicates no statistically significant difference between the 2 groups.

### Risk factors for hardware-related complications

To identify potential predictors of hardware failure, a comparative analysis was performed between the complication group (n = 5) and the non-complication group (n = 41).

Univariate analysis revealed that preinjury activity level was a significant risk factor for hardware-related issues. Specifically, 80% (4/5) of patients in the complication group were categorized as having a high activity level, compared to only 24.4% (10/41) in the non-complication group (*P* = .018, Fisher's exact test). While statistically significant within our cohort, this finding should be interpreted as a preliminary observation given the small number of events.

Regarding age, the mean age of the complication group was 42.0 ± 14.5 years, while the non-complication group had a mean age of 50.2 ± 11.2 years; this difference did not reach statistical significance (*P* = .156).

Bone quality also played a role in early failure; the only case of fixation failure occurred in a patient with diagnosed osteoporosis. However, across the entire cohort, osteoporosis was not a statistically significant predictor of overall hardware failure (*P* = .109, Fisher's exact test). Other factors, including dislocation direction (anterior vs. posterior) and operative time, showed no significant correlation with the occurrence of complications (all *P* > .05) (Table IV).

**Table IV:** Comparison of baseline and clinical characteristics between complication and non-complication groups

Variables	Complication group (n = 5)	Non-complication group (n = 41)	*P* value
Age (yrs)	42.0 ± 14.5	50.2 ± 11.2	.156∗
Gender (male/female)	5/0	39/2	>.999†
High activity level, n (%)	4 (80%)	10 (24.4%)	.018†
Osteoporosis, n (%)	1 (20%)	2 (4.9%)	.109†
Posterior dislocation, n (%)	1 (20%)	3 (7.3%)	.384†
Operative time (min)	68.2 ± 9.5	64.8 ± 8.2	.412∗
Timing of removal (mo)	8.8 ± 1.7	8.2 ± 2.1	.560∗

∗ Calculated using the Mann-Whitney U test.

† Calculated using Fisher's exact test. Bold value indicates statistical significance (*P* < .05).

### Radiographic severity and outcome correlation

The mean pre-operative displacement of the proximal clavicle was 24.8 ± 5.2 mm (range: 15-38 mm). To determine whether the severity of the initial injury influenced functional recovery or mechanical stability, Spearman correlation analysis was performed (Table V). No statistically significant relationship was observed between the magnitude of initial displacement and final CSSs (ρ = −0.124, *P* = .412) or VAS scores for pain (ρ = 0.086, *P* = .568). Similarly, the severity of pre-operative displacement was not significantly correlated with the occurrence of hardware-related complications (ρ = 0.152, *P* = .315). These findings suggest that the clinical efficacy of the SC hook plate in restoring joint function is independent of the initial degree of clavicular displacement.

**Table V:** Spearman correlation analysis between pre-operative displacement and clinical outcomes (n = 46)

Variable 1	Variable 2	Correlation coefficient (*ρ*)	*P* value
Pre-operative displacement (mm)	Post-operative CSS	−0.124	.412
Pre-operative displacement (mm)	Post-operative VAS	0.086	.568
Pre-operative displacement (mm)	Hardware-related complications∗	0.152	.315

*CSS*, Constant Murley Shoulder Score; *VAS*, visual analog scale; *CT*, computed tomography.

Pre-operative displacement was defined as the maximum distance (mm) of clavicular migration relative to the sternal notch measured on axial CT scans. The ρ (Spearman's Rho) coefficient represents the strength and direction of the association between variables. Hardware-related complications (comprising plate breakage and fixation failure) were analyzed as a categorical variable (presence vs. absence). Statistical significance was defined as a 2-tailed *P* value < .05. The lack of a significant correlation (*P* > .05) indicates that the clinical efficacy of the sternoclavicular hook plate is independent of the initial magnitude of joint displacement.

∗ Hardware-related complications include plate breakage and early fixation failure, coded as a binary categorical variable (1 = complication present, 0 = no complication) for Spearman correlation analysis.

## Discussion

This study contributes a systematic evaluation of an SC hook plate specifically designed for the surgical treatment of traumatic SCJ dislocations, augmenting the emerging literature on this fixation modality. While various internal fixation strategies have been described, our investigation provides novel insights into the clinical performance and mechanical behavior of a dedicated sternal hook‑clavicular plate construct within a consistent cohort of traumatic injuries. Our findings align with recent reports by Guo et al^7^ and Xin et al,^20^ who demonstrated that hook plates can achieve reliable stabilization in SCJ injuries. However, several distinctions characterize the present series. Unlike the work by Qu et al,^15^ which used adapted acromioclavicular hook plates for anterior dislocations, our study evaluates a system anatomically contoured specifically for the SCJ anatomy. Furthermore, while Guo et al^7^ reported on a heterogeneous group including both dislocations and fractures at a Level I trauma center, our study specifically isolates pure dislocations to characterize the temporal pattern of hardware failure. This specific focus reveals a dissociation between mechanical implant integrity and functional success—a finding that complements and extends the current understanding of SCJ stabilization.^20^

In the present cohort, significant improvements in clinical outcomes were observed following surgical stabilization. The mean CSS increased from 36.4 ± 4.8 pre-operatively to 86.6 ± 6.2 at final follow-up, and VAS pain scores improved from 7.2 ± 1.4 to 1.6 ± 0.4, supporting the effectiveness of internal fixation in restoring shoulder function. Rigid fixation strategies, including plate use, have been shown to restore stability in shoulder injuries, but retention duration and soft-tissue healing remain important determinants of functional recovery.^9^^,^^11^^,^^12^

Despite these favorable outcomes, hardware-related complications occurred in 10.9% of patients, including one early fixation failure (2.2%) and 4 cases of asymptomatic plate breakage (8.7%). Although hardware failure has been infrequently reported in other SCJ fixation modalities, the unique biomechanical environment of the SCJ—characterized by triaxial motion and dependence on soft-tissue stability—poses challenges for rigid fixation across a mobile joint.^6^^,^^13^ Additionally, applications of hook plates in complex shoulder girdle injuries have highlighted the potential for mechanical issues under cyclic loading conditions.^14^

This consistent temporal and morphological pattern suggests that plate breakage may reflect a fatigue-driven mechanical process rather than technical failure or acute overload. The SCJ is inherently a highly mobile articulation, undergoing complex multidirectional motion during physiological shoulder activity.^8^ During the early post-operative phase, the hook plate provides necessary rigid stabilization while capsuloligamentous healing progresses. It is hypothesized that once sufficient biological stability is restored, continued rigid fixation across this dynamic joint exposes the implant to repetitive cyclic loading. Over time, these cumulative stresses concentrate at the hook-plate junction—a recognized stress riser—potentially exceeding the fatigue endurance of the implant material.^15^

Within this framework, late plate breakage could represent a possible mechanical consequence of prolonged retention in an active joint. This interpretation is supported by the preserved joint congruity and favorable functional outcomes observed in all patients, which may imply that intrinsic soft-tissue healing had already assumed the primary stabilizing role by the time mechanical discontinuity occurred,^3^ although direct histological or imaging-based confirmation of ligamentous integrity was not performed.

Univariate analysis in our cohort demonstrated a significant association between high preinjury activity levels and the occurrence of hardware-related complications (*P* = .018). In accordance with established biomechanical principles, patient activity level likely functions as an accelerator of cyclic loading, increasing rather than a direct cause of acute failure.^17^ Increased frequency and intensity of shoulder motion in highly active individuals—such as manual laborers or athletes—enhance the rate of fatigue accumulation within rigid implants placed across mobile articulations. This kinetic interpretation is consistent with observations in shoulder trauma literature, where younger age and higher functional demands are often associated with increased rates of hardware-related complications under repetitive stress.^16^ While we did not perform direct biomechanical testing, our clinical findings suggest a potential trend where activity level modulates the temporal progression toward mechanical exhaustion in the SC hook plate.

Importantly, the dissociation between mechanical implant integrity and clinical outcome highlights the transitional role of the SC hook plate. Once biological healing is complete, the implant’s structural integrity may become less critical for maintaining joint stability, as its primary stabilizing role may have transitioned to the healed soft tissues. Residual implant components may continue to provide passive restraint against gross translation, further contributing to maintained reduction. These observations support conceptualizing the SC hook plate as a temporary biomechanical scaffold designed to protect the joint during the vulnerable healing phase.^3^

Our temporal observations revealed that all instances of plate breakage in this series occurred in patients who retained the implant for more than 6 months. Consequently, elective hardware removal within this timeframe could be considered as a potential strategy to mitigate the risk of fatigue-related failure. However, this clinical consideration is derived from a limited number of events (n = 4) and must be interpreted with significant caution. It is important to acknowledge that our study does not provide comparative data evaluating the functional or radiographic outcomes of early vs. late removal protocols. Therefore, while elective removal around 6 months appears biomechanically plausible to prevent fatigue breakage, further prospective comparative studies are required to validate this approach and establish definitive clinical guidelines.

Several additional limitations must be acknowledged to provide a balanced interpretation of our findings. First, a central constraint of our study is the absence of any validated measure of biological healing at the time of plate breakage. While we inferred that ligamentous stability had been achieved based on the absence of redislocation, we did not perform histological assessments or specialized imaging (such as dynamic magnetic resonance imaging) to confirm the structural integrity of the capsuloligamentous complex. Second, our cohort was predominantly male (95.7%), which significantly limits the generalizability of these results to female patients. Women may exhibit differences in bone mineral density, baseline activity levels, and soft-tissue healing characteristics that were not captured in this series. Third, and most importantly, the very small number of hardware-related complications (n = 5) severely limits the statistical power and interpretability of our risk factor analysis. This small sample size represents a fundamental constraint on the conclusions that can be drawn regarding predictors of failure. Consequently, the identified association between activity level and breakage should be viewed as preliminary and hypothesis-generating rather than a definitive clinical rule. Finally, the retrospective design and absence of a control group managed with alternative fixation methods preclude direct comparative efficacy assessments.

In summary, this large single-center clinical series of SC hook plate fixation demonstrates effective restoration of function in traumatic SCJ dislocations while revealing a characteristic pattern of time-dependent implant fatigue. These findings suggest that mechanical breakage represents an expected endpoint once biological stability has been achieved rather than a failure of surgical stabilization. Recognizing the SC hook plate as a temporary internal splint may guide implant retention strategies, patient counseling, and future device development. Prospective studies incorporating biomechanical modeling and comparative treatment arms are warranted to validate these hypotheses and refine evidence-based management of unstable SCJ dislocations.^6^

## Conclusion

SC hook plate fixation effectively restores shoulder function and maintains anatomic joint stability in patients with traumatic dislocations. While clinical and radiographic outcomes are favorable, the procedure is associated with a notable rate of hardware complications, primarily characterized by late asymptomatic plate breakage and occasional early fixation failure. A high preinjury activity level represents a significant clinical risk factor for these mechanical events. Importantly, the occurrence of late implant fatigue does not appear to compromise final functional recovery or joint reduction. Based on our findings, the SC hook plate functions effectively as a temporary stabilizer; therefore, elective hardware removal within 6 months post-operatively is a prudent strategy to mitigate the risk of fatigue-driven failure, especially in physically active individuals.

## Disclaimers:

Funding: No funding was disclosed by the authors.

Conflicts of interest: The authors, their immediate families, and any research foundations with which they are affiliated have not received any financial payments or other benefits from any commercial entity related to the subject of this article.

## References

[1] Apostolakos J.M., Jildeh T.R., Dey Hazra R.O., Dey Hazra M.E., Chang P.S., Geissbuhler A.R. (2023). Sternoclavicular joint reconstruction with Gracilis Tendon Autograft. Arthrosc Tech.

[2] Barbaix E., Lapierre M., Van Roy P., Clarijs J.P. (2000). The sternoclavicular joint: variants of the discus articularis. Clin Biomech (Bristol).

[3] Cai G.P., Xu C.L., Deng B., Hong H.X., Liang J.B., Lin L. (2021). Novel sternoclavicular hook-plate for treatment of proximal clavicle fracture with dislocation of sternoclavicular joint. Zhongguo Gu Shang.

[4] Ciatti C., Masoni V., Maniscalco P., Asti C., Puma Pagliarello C., Caggiari G. (2024). Management options for traumatic posterior sternoclavicular joint dislocation: a narrative review with a single institution's experience. J Clin Med.

[5] Cutteridge J., Dixon J., Garrido P., Peckham N., Smith C., Woods A. (2025). A systematic review and meta-analysis of operative versus non-operative management for first time traumatic anterior shoulder dislocation in young adults. Shoulder Elbow.

[6] Dhawan R., Singh R.A., Tins B., Hay S.M. (2018). Sternoclavicular joint. Shoulder Elbow.

[7] Guo P., Li N., Zhong H., Zhao G., Pan Z. (2025). Therapeutic effect of the sternoclavicular hook plate in severe trauma patients with sternoclavicular joint injuries: from a level-Ⅰ trauma center. World J Emerg Med.

[8] Kalantar S.H., Bagheri N., Bidaki M., Vosoughi F. (2021). Superior sternoclavicular joint dislocation presented with shoulder motion limitation: a case report and literature review. Int J Surg Case Rep.

[9] Kim K.B., Lee Y.S., Wang S.I. (2023). Clinical outcome after clavicular hook plate fixation for displaced medial-end clavicle fractures. Clin Orthop Surg.

[10] Lacheta L., Dekker T.J., Goldenberg B.T., Horan M.P., Rosenberg S.I., Pogorzelski J. (2020). Minimum 5-Year clinical outcomes, survivorship, and return to sports after hamstring Tendon autograft reconstruction for sternoclavicular joint instability. Am J Sports Med.

[11] Lee S.J., Eom T.W., Hyun Y.S. (2022). Complications and frequency of surgical treatment with AO-Type hook plate in shoulder trauma: a retrospective study. J Clin Med.

[12] Lin H.Y., Wong P.K., Ho W.P., Chuang T.Y., Liao Y.S., Wong C.C. (2014). Clavicular hook plate may induce subacromial shoulder impingement and rotator cuff lesion--dynamic sonographic evaluation. J Orthop Surg Res.

[13] Morell D.J., Thyagarajan D.S. (2016). Sternoclavicular joint dislocation and its management: a review of the literature. World J Orthop.

[14] Nie P., He X., Zhang Z., Chen P., Wang R., Fang Q. (2026). Optimizing clavicle hook plate fixation through biomechanical analysis of pre-bent plate condition and screw configurations. Front Med (Lausanne).

[15] Qu Y.Z., Xia T., Liu G.H., Zhou W., Mi B.B., Liu J. (2019). Treatment of anterior sternoclavicular joint dislocation with acromioclavicular joint hook plate. Orthop Surg.

[16] Sarangi P. (2025). Shoulder replacement in the under 55's is anatomical or reverse the best solution?. EFORT Open Rev.

[17] Tardioli A., Malliaras P., Maffulli N. (2012). Immediate and short-term effects of exercise on tendon structure: biochemical, biomechanical and imaging responses. Br Med Bull.

[18] Wang S., Chen Z., Lin L., Pan Q., Wang B., Liu F. (2021). Long-term results for traumatic sternoclavicular joint dislocation treated with a sternoclavicular joint-specific plate. ANZ J Surg.

[19] Wu K., Li B., Guo J.J. (2021). Fatigue crack growth and fracture of internal fixation materials in in vivo Environments-A review. Materials (Basel).

[20] Xin H., Wang X., Zhang S., Lin L., Chen H., Hong H. (2023). Novel sternoclavicular hook plate for the treatment of posterior sternoclavicular dislocation: a retrospective study. J Orthop Surg Res.

